# Remote Testing of Reading Comprehension in 8-Year-Old Children: Mode and Setting Effects

**DOI:** 10.1177/10731911231159369

**Published:** 2023-03-08

**Authors:** Timo Gnambs, Wolfgang Lenhard

**Affiliations:** 1 Leibniz Institute for Educational Trajectories, Bamberg, Germany; 2 University of Würzburg, Germany

**Keywords:** reading comprehension, computerized testing, mode effect, measurement bias

## Abstract

Proctored remote testing of cognitive abilities in the private homes of test-takers is becoming an increasingly popular alternative to standard psychological assessments in test centers or classrooms. Because these tests are administered under less standardized conditions, differences in computer devices or situational contexts might contribute to measurement biases that impede fair comparisons between test-takers. Because it is unclear whether cognitive remote testing might be a feasible assessment approach for young children, the present study (*N* = 1,590) evaluated a test of reading comprehension administered to children at the age of 8 years. To disentangle mode from setting effects, the children finished the test either in the classroom on paper or computer or remotely on tablets or laptops. Analyses of differential response functioning found notable differences between assessment conditions for selected items. However, biases in test scores were largely negligible. Only for children with below-average reading comprehension small setting effects between on-site and remote testing were observed. Moreover, response effort was higher in the three computerized test versions, among which, reading on tablets most strongly resembled the paper condition. Overall, these results suggest that, on average, even for young children remote testing introduces little measurement bias.

Digital media, such as laptops, tablets, or smartphones, increasingly shape psychological assessments (e.g., Steger et al., 2019; Wright, 2020; Zinn et al., 2021). Particularly, for cognitive measurements in educational contexts computerized proficiency testing has become the *de facto* standard in many applied areas. For example, many high-stake college admission or proficiency certification procedures adopt computerized testing formats (e.g., Hurtz & Weiner, 2022; Steedle et al., 2022) because they allow for better standardization of test instructions, item presentations, and response coding, thus, leading to less error-prone and fairer measurements. Even many educational large-scale studies have recently switched to computers as their preferred medium of assessment that also allows for administering innovative item formats (e.g., simulation-based items) and collecting ancillary information (e.g., process data) to capture novel constructs more precisely (see von Davier et al., 2019).

In recent years, psychological assessments have faced another significant shift. Often, they had to be conducted remotely (over the internet) in the private homes of the test-takers without the physical presence of a supervisor (e.g., Cherry et al., 2021; Hurtz & Weiner, 2022; Papageorgiou & Manna, 2021) because traditional on-site testing in dedicated test centers or classrooms was not feasible for economic or health reasons. Although these tests can be proctored in one way or another, for example, by human supervisors via video and screen sharing or by artificial intelligence systems that automatically analyze test-takers’ computer activities or video captures to detect suspicious activities (Langenfeld, 2022), these procedures are typically characterized by substantially less restrictive control over the test setting. Rather, differences in environmental conditions (e.g., lighting, computer devices), distractions (e.g., noise, people entering the room), or inadmissible support by parents and unauthorized aids can threaten the comparability of cognitive assessments at home (e.g., Bridges et al., 2020; Dendir & Maxwell, 2020; Passell et al., 2021). However, standardized assessment procedures are a prerequisite to interpreting performance differences on psychological tests in terms of individual differences between test-takers (Flake & Fried, 2020; Schroeders & Gnambs, 2020).

So far, findings on remote cognitive testing are dominated by research on adolescents and (young) adults, often from clinical populations (e.g., Cherry et al., 2021; Guo, 2022; Hurtz & Weiner, 2022; Kim & Walker, 2021; Leong et al., 2022; Segura & Pompéia, 2021). Whether remote testing might also represent a viable approach for young children is as of yet largely unexplored territory. Therefore, the present study evaluated the measurement equivalence of a validated test of basic reading comprehension in German (ELFE-II; Lenhard, Lenhard, & Schneider, 2017) that was administered to a sample of 8-year-old children in a remote setting in their private homes or an on-site setting at school. In contrast to previous research, we tried to disentangle mode and setting effects to highlight to what degree differences in the test setting or a switch from paper to computerized testing contributed to a potential non-comparability of remote testing.

## Components of Reading Comprehension

Reading abilities represent essential skills for successful participation in modern societies. Proficient reading abilities are not only important prerequisites to succeed in educational and occupational contexts (e.g., Spengler et al., 2018), but also shape the development of other domain-specific competences, such as mathematics (Gnambs & Lockl, 2022). Therefore, the acquisition of appropriate levels of reading comprehension is a central goal in primary school. Reading requires multiple cognitive processes that can be viewed as a hierarchical system (Ahmed et al., 2014). On the most basic level, this includes the fast and accurate decoding of words and the syntactic parsing of sentences to establish local coherence (Cain & Oakhill, 2011; Schindler & Richter, 2018). In contrast, on higher hierarchical levels, reading comprehension requires the ability to integrate information contained in single words and sentences into a coherent overall picture of a text, the so-called situation model (van Dijk & Kintsch, 1983). Reading proficiency is thus characterized by the ability to integrate facets of information and to reconstruct the encoded meaning, to enrich this meaning by prior knowledge and to draw inferences that supplement or continue the information presented in a text. Therefore, modern instruments for the measurement of reading competence adopt a multi-process perspective capturing reading on the word, sentence, and text level (e.g., Lenhard, Lenhard, & Schneider, 2017).

## Characteristics of Remote Testing

Remote testing represents a mixture of different test-taking conditions (see Kroehne, Gnambs, & Goldhammer, 2019). Most notably, it involves a mode switch from traditional paper-based tests that dominated educational assessments for decades to computerized administration formats. Moreover, it often also refers to unsupervised and unstandardized assessments because it can be conducted without the presence of a test administrator in highly variable settings in the test-takers private homes. Each of these factors or their combination might result in systematically distorted measurements that can prevent fair comparisons between test-takers. So far, the most unambiguous findings are available regarding the presence of a test supervisor during the assessment. Meta-analytic evidence (Steger et al., 2020) highlights that test-takers are more likely to cheat in unsupervised settings (e.g., searching correct answers on the internet) resulting in significantly higher test scores as compared with situations supervised by test administrators, independent of potential-counter measures that were implemented to deter cheating. Therefore, most remote tests of cognitive abilities implement some form of supervision, particularly in high-stake contexts. In contrast, the available findings on mode and setting effects are less clear.

### Mode Effects for Tests of Reading Comprehension

A plethora of studies suggested that the switch from traditional paper-based to computerized assessment formats has, on average, a negligible impact on test results of power tests (e.g., Schroeders & Wilhelm, 2011; Zinn et al., 2021). Although respective mode effects are often small, early meta-analyses suggested that they might depend on different factors, such as the measured construct or the target population (e.g., Kingston, 2009; Wang et al., 2007, 2008). Particularly for tests of reading comprehension, more recent investigations with substantially larger and heterogeneous samples led to a more ambivalent picture. For example, 15-year-old students performed significantly worse on the Program for International Student Assessment (PISA) reading tests when administered on a computer as compared with paper (Jerrim et al., 2018; Robitzsch et al., 2020). A similar pattern was also reported for mandatory state-wide student performance evaluations in Germany (Wagner et al., 2022) that resulted in lower reading test performance on computerized test versions among eighth graders, particularly for low-achieving students. Other studies replicated these results for younger age groups, such as 10- to 13-year-old children (Golan et al., 2018; Kerr & Symons, 2006; Støle et al., 2020). Based on these findings, several meta-analyses on mode effects in reading performance (Clinton, 2019; Delgado et al., 2018; Kong et al., 2018) found, on average, lower scores in computerized testing with pooled effects corresponding to Cohen’s *d*s between –0.25 and -0.54.

Despite numerous studies on mean-level differences between different administration modes, not all findings agree on substantial mode effects for all tests and samples (e.g., Porion et al., 2016; Rockinson-Szapkiw et al., 2013). Some authors also found evidence for construct equivalence between the two assessment modes (Kroehne, Buerger, et al., 2019), thus, giving little support for digital reading as a distinct construct from paper-based reading. Even for the ELFE-II test, approximate measurement invariance across paper-based and computerized test versions could be established for first to sixth graders (Lenhard, Schroeders, & Lenhard, 2017); albeit children produced slightly more errors on the computer.

The reasons underlying the observed mode effects are still debated. Some authors argued that mode effects are item-specific and depend on certain item properties, such as response formats or item ordering (see Buerger et al., 2019). As a result, the switch to computerized administrations should not affect the entire test but only selected items. In line with this conjecture, only six of 35 items in a test of reading comprehension showed significant mode effects in a sample of 15-year-old adolescents (Kroehne, Buerger, et al., 2019). Others proposed differences in test-taking behavior as a potential explanation. For example, test-takers tend to take less time on the computer and finish tests quicker, while showing higher guessing behavior, particularly among low-performance students (Karay et al., 2015; Singer et al., 2019); albeit also opposite results were sometimes observed (Steedle et al., 2022). An experimental study that independently varied the presentation medium (paper versus computer) of the reading text and the test items suggested that the mode effect in reading comprehension is primarily a result of digital reading rather than a media-induced testing effect (Ben-Yehudah & Eshet-Alkalai, 2021). Consequently, different cognitive explanations have been put forward for the inferiority of digital text comprehension. For example, some authors suggested that the light emitted by digital media might contribute to visual fatigue and, consequently, increases cognitive load (Benedetto et al., 2013), while others emphasized inferior learning strategies that people adopt on digital devices, thus, resulting in higher reading speed but shallower processing of the reading material (e.g., Isaacson, 2017; Morineau et al., 2005; Singer et al., 2019). Alternatively, it has also been suggested that people are more overconfident about their performance when reading on a computer that might lead to poorer test results (Ackerman & Goldsmith, 2011). Finally, mode effects might also be a consequence of respondents’ limited access to and experience with computers that can result in poorer digital skills (see the review by Lynch, 2022). For example, higher computer familiarity tends to be associated with higher scores on computerized assessments (Bennett et al., 2008; Chan et al., 2018), as long as open response formats were part of the test. In contrast, for tests with simpler item formats (e.g., multiple-choice), differences in computer skills hardly affect mode differences in test performance (Higgins et al., 2005).

Taken together, the available findings suggest small mode effects in tests for reading comprehension disadvantaging computerized assessments. However, the size of these effects seems to vary depending on the administered test and the examined sample. So far, only a few studies examined mode effects in tests of reading performance for young children (Golan et al., 2018; Kerr & Symons, 2006; Lenhard, Schroeders, & Lenhard, 2017; Støle et al., 2020).

### Setting Effects in Remote Testing

While standard psychological assessments are typically conducted by administering tests under highly controlled conditions to ensure that they are consistent for all test-takers (e.g., on comparable computers in dedicated test centers), remote testing places the burden of standardization on the test-taker. Although test administrators can recommend optimal testing conditions, in practice, different technological devices (e.g., laptops, tablets, smartphones) will be used by test-takers in different situational contexts (see Davis, 2015; Leeson, 2006). These differences might involuntarily limit the comparability of measurements. For example, different input devices, such as a touchscreen or a mouse, can affect performance on computerized tasks (e.g., Cockburn et al., 2012). More importantly, these differences might be moderated by characteristics of the test-taker, such as age or computer experience (Findlater et al., 2013). Although device effects are more pronounced for timed assessments (e.g., Bridges et al., 2020; Passell et al., 2021), differences in, for example, screen size or resolution might also affect untimed power tests, particularly if they require reading or discerning complex stimuli (Bridgeman et al., 2003). In addition to technological variations, the test situation might not be equally controllable resulting in distractions, such as disturbing noise or people entering the room. This might be particularly problematic for young children with still-developing self-regulative abilities, for which upholding sustained attention might be particularly challenging. Prevalence estimates of test-takers experiencing environmental distractions while taking a web-based cognitive test vary between 7% and 33% (Backx et al., 2020; Madero et al., 2021). However, so far, it is unclear whether this rate is substantially larger than in, for example, group-based testing in classrooms and, more importantly, whether these distractions have a meaningful impact on test performance. Initial studies comparing historical data from computerized licensure programs administered in test centers to proctored web-based tests, so far, found only negligible differences between the two assessment settings (Cherry et al., 2021; Hurtz & Weiner, 2022; Kim & Walker, 2021). Altogether, there is still rather limited systematic research on setting effects in psychological cognitive testing. Moreover, most research refers to (sometimes highly selective) adolescent and adult samples. Little is known whether young children with still-developing self-regulative abilities (see Montroy et al., 2016, for respective longitudinal trajectories) might be more susceptible to device effects or environmental distractions and, thus, experience remote testing as more challenging.

## Objectives of the Present Study

Remote cognitive testing might develop into a valuable alternative to traditional psychological assessment if comparable psychometric properties can be established and mode or setting effects do not systematically distort measurements. Prior research on different aspects of remote testing often relied on rather small and selective samples; for example, the median sample size in a meta-analysis of mode effects in reading performance was 67 (Delgado et al., 2018). More importantly, with notable exceptions (Golan et al., 2018; Kerr & Symons, 2006; Lenhard, Schroeders, & Lenhard, 2017; Støle et al., 2020), they primarily focused on adolescents and adults, but rarely addressed young children. Therefore, the present study examined the feasibility of testing reading comprehension of over 1,500 German children at the age of 8 years in a remote setting. In contrast to most previous research, we tried to disentangle different sources of potential measurement bias by making use of a quasi-experimental design that tested children either on paper or a computer device (mode effect) remotely at home or on-site at school (setting effect). Furthermore, potential device effects were examined by presenting the remote test version either on a tablet or laptop. Based on the available research summarized above, we expected the following effects: (a) Paper-based assessments were assumed to result in higher reading performance as compared with computer-based assessments (Clinton, 2019; Delgado et al., 2018; Kong et al., 2018). (b) Although prior research in adult samples suggested only negligible setting effects (Cherry et al., 2021; Hurtz & Weiner, 2022; Kim & Walker, 2021), it is conceivable that environmental distractions in remote settings might lead to poorer test performance for children. (c) Because we administered a power test without a high degree of speededness, substantial device effects were not expected. To this end, analyses of differential response functioning (DRF) were conducted to evaluate the psychometric properties of the ELFE-II test (Lenhard, Lenhard, & Schneider, 2017) and how these might be affected by different assessment conditions.

## Method

### Participants

Mode and setting effects were examined by combining two independent samples from a remote and an on-site assessment. The remote sample was part of the longitudinal *National Educational Panel Study* (NEPS; Blossfeld & Roßbach, 2019) that follows multiple age cohorts across their life courses. We focus on the newborn cohort that was initially drawn using a stratified cluster sampling design to cover children born in Germany between January and June 2012 (see Aßmann et al., 2019). The most recent assessment included *N* = 1,319 children attending Grade 2 in primary schools from all German federal states. We excluded children with diagnosed dyslexia, attention deficit hyperactivity disorder, or special educational needs (*n* = 69) and students that had repeated a class (*n* = 3). Because we were interested in examining unambiguous device effects the sample was further limited to children using a tablet (with a touchscreen) or a laptop (with a mouse), thus, excluding *n* = 64 additional children that used a laptop with a touchpad. This resulted in an analysis sample of 1,183 children (51% girls) with a mean age of 8.26 years (*SD* = 0.12). About 81% of them reported speaking German at home. Most children (*n* = 998) worked on tablets, while the rest of them (*n* = 185) worked on laptops (see Table 1). All children were tested in the last 2 months of second grade in primary school (i.e., School Months^1^ 10 or 11) or during their summer vacation before entering third grade (i.e., School Month 12).

**Table 1.** Sample Characteristics Across Assessment Groups.

	Remote samples			On-site samples		
Sample characteristic	Total	Tablet	Laptop	Total	Computer	Paper
Sample size	1,183	998	185	407	200	207
Unweighted original samples						
Percentage girls	51	52	48	52	54	50
Mean age in years (*SD*)	8.26 (0.12)	8.26 (0.12)	8.25 (0.13)	8.34 (0.34)	8.32 (0.35)	8.35 (0.34)
Percentage German spoken	81	82	80	70	68	72
Mean school month (*SD*)	11.23 (0.68)	11.21 (0.68)	11.29 (0.65)	10.28 (1.09)	10.30 (1.14)	10.25 (1.04)
Weighted balanced samples						
Percentage girls	51	52	52	51	50	53
Mean age in years (*SD*)	8.26 (0.00)	8.26 (0.00)	8.26 (0.00)	8.28 (0.02)	8.28 (0.02)	8.28 (0.02)
Percentage German spoken	79	79	79	76	77	75
Mean school month (*SD*)	11.18 (0.46)	11.18 (0.46)	11.18 (0.46)	10.96 (1.93)	10.94 (2.14)	10.98 (1.74)

*Note*. The school month refers to the number of months since the beginning of the current school year (see Lenhard, Lenhard, & Schneider, 2017). Because the beginning of the school year slightly differs between the German federal states, the same school month might refer to different months of the year.

The on-site sample was part of the norm data for the revised reading comprehension test ELFE-II (Lenhard, Lenhard, & Schneider, 2017)^2^ that included *N* = 502 children from nine federal states in Germany attending primary schools at the end of second grade (i.e., School Months 9–11) and the beginning of third grade (i.e., School Month 1). To more closely match the age range of the remote sample, we excluded *n* = 68 children falling outside the age range of 7.5 to 9.0 years. Because children with dyslexia or special educational needs (*n* = 11) and children who repeated a class (*n* = 16) were also excluded from the present analyses, the analysis sample comprised 407 children (52% girls). They had a mean age of 8.34 years (*SD* = 0.34) and about 70% of them indicated speaking German at home. About half of the children (*n* = 207) worked on a paper-based test, while the rest (*n* = 200) worked on a computerized version of the same test (see below).

### Procedures and Administration Settings

The remote assessment was conducted in the summer of 2020 by professional test administrators from a survey institute at the private homes of the children. A couple of weeks before, the assessment the necessary computer equipment in the household was evaluated in a telephone interview. Although tablets were preferred, laptops with a minimum screen size were allowed as alternative assessment devices. If the available devices allowed the child to take the remote test, the test administrator called the parent by phone at the prearranged test date to assist in setting up the tablet or laptop (e.g., positioning the device on the table) and starting the web-based test (e.g., opening the browser, entering the correct link and password). Then, the parent was asked to leave the room to let the child work alone on the remote test. During the test administration, the test administrators supervised the child’s progress on the test remotely using a dashboard that showed in real time the test page a child was currently visiting. Assistance and verbal support to the child were provided by phone. Thus, the test administrator had a continuous means of communication with the child during the entire test procedure. Although the test administrator could not directly see the child or the specific testing conditions, such as the room a child, was occupying or whether other people were present during the assessment, they could monitor the child’s progress in the test, listen to voiced problems or background noise, and talk to the child. Though, direct assistance through test administrators was rarely required by design because the remote test used video instructions that introduced the tasks and, thus, allowed a high level of standardization. The role of the test administrators was primarily limited to assisting in starting the test, motivating children between different tests, and helping with unforeseen problems during the test. The reading comprehension test was embedded in a test battery including different cognitive tests and was always presented second after finishing a test of reading speed with a length of 2 minutes.

The on-site data were collected in 2015 by trained undergraduates in different schools. At school, the children were divided into smaller groups of up to eight (for the computer condition) or 25 students (for the paper condition). Then, the children worked individually on the test while the supervisors were continually present in the room to monitor the children and provide support in case of difficulties.

### Instrument and Administration Modes

Reading comprehension was measured with the ELFE-II test (Lenhard, Lenhard, & Schneider, 2017) which is a widely used measure of reading performance in German for children from first to seventh grades. Although the test includes three subtests measuring reading comprehension on the word level, sentence level, and text level, the current study only administered the text level subtest. The subtest presents several short texts (including two to eight sentences) that are accompanied by one to three items. Each of the 26 multiple-choice items includes four response options with one being correct and three response options functioning as distractors (i.e., they are incorrect). Following established models of text comprehension (Zwaan & Singer, 2003), the theoretical construction rationale of these items specified three independent factors. The text addressed by each item presents either a fictional or a non-fictional topic (factor genre: non-fiction versus fiction) that requires retrieving a literal piece of information or drawing an analogy from the presented information (factor information: literal versus analogous). Moreover, each item requires either drawing connections between neighboring sentences or between multiple sentences (factor coherence: local versus global). The items cover all combinations of the three factors to measure a unidimensional reading comprehension construct. The items are roughly ordered by their difficulty with easier items at the beginning of the test and more difficult items at the end of the test. The subtest features good reliability of *r_tt_* = .85 after a retest interval of 1 month, corresponds well with the overall subjective teacher rating of children’s reading abilities (*r* = .64), with other tests on reading proficiency, and it has been systematically evaluated regarding the effects of sex, language background, and learning disorders (Lenhard, Lenhard, & Schneider, 2017).

In the remote setting, the children used their private computers to work on the test. Most children interacted with the assessment device by touch on a tablet, while a subsample used laptops that required mouse interactions (see Table 1). In the on-site setting, the computerized tests were administered on the technical equipment in the respective schools and, thus, consisted of different types of personal computers that used a mouse as an input device. In all administration conditions, the children received the same instructions. In the remote and on-site computer conditions, the children worked individually on the practice items and also received automatic feedback from the testing environment, whereas in the on-site paper condition the instructions were presented by the supervisors. Each item was presented on an individual page and, in the remote and computer conditions, did not require scrolling. In all conditions, the children received the identical item content in the same order and had to finish the test within 7 minutes.

The reliability estimates fell at .88 and .88 in the remote tablet and laptop conditions, while the respective values were .81 and .90 for the on-site computer and paper conditions, thus, indicating no pronounced reliability differences between the four assessment groups. Because of the time limit, many children did not finish all items of the test. Following the scoring instructions in the works of Lenhard, Lenhard, & Schneider (2017), missing values were scored as incorrect responses. However, we also calculated the number of answered items (correct and incorrect responses) as an indicator of response effort that has a theoretical range from 0 to 26.

### Statistical Analyses

#### Item Response Modeling

Following the scoring scheme outlined in the test manual (Lenhard, Lenhard, & Schneider, 2017), a one-parametric item response model (Rasch, 1960) was fitted to the item scores using marginal maximum likelihood estimation. To place the measurements in the four administration conditions on a common scale, we used a multi-group item response model with invariance constraints on selected anchor items. The anchor items were identified following Woods (2009) by first estimating a fully unrestricted multi-group model. The population means and variances in the remote tablet condition (i.e., the reference group) were fixed to 0 and 1, respectively, for model identification, while the respective parameters were freely estimated in the other groups. Then, item difficulties were freed across groups’ one item at a time. Model comparisons between the fully restricted model and the less restricted models using likelihood ratio tests with Benjamini and Hochberg’s (1995) correction identified five anchor items with measurement invariant parameters across assessment conditions.

Model comparisons examined whether mode and setting effects were item-specific or homogeneous across all items. To this end, the linked multi-group model was compared with a model that additionally placed equality constraints on the remaining item parameters and only allowed for latent-mean differences. A superior fit of the latter would indicate homogeneous differences between the four assessment groups because potential mode or setting effects are absorbed in the latent means.

#### Differential Response Functioning

Mode and settings effects between the remote tablet and laptop conditions, and the two on-site conditions with computer or paper administrations were analyzed by examining DRF for single items and the entire test. A test exhibits differential item or test functioning (DIF, DTF) when the expected item or test scores differ between groups although the latent proficiency is held constant (Millsap, 2011; Penfield & Camilli, 2007). For example, in case, sex significantly predicts the outcome of an item above the estimated ability of a person, then the difficulty is different for males and females. If the effect is constant across all ability levels, this is called a uniform DIF. In case, the effect additionally interacts with the ability (non-uniform DIF), then persons of one sex with low ability would perform even more poorly in this item, then expected, whereas highly proficient persons of that sex would have an increased change of succeeding. DIF is often used to assess test fairness and comparable analyses can not only be applied to single items, but also to complete scales. Thus, DIF examines biases in item parameters, whereas DTF evaluates how biases accumulate across items and leads to biased test scores for the comparison of groups.

DIF and DTF were quantified following the work of Chalmers (2018) based on the linked multi-group model by calculating the differences in the item and test score functions between the remote tablet condition and each of the three other conditions. These differences are captured by the compensatory DRF statistics cDIF (*compensatory differential item functioning*) and cDTF (*compensatory differential test functioning*) that represent the condition-specific biases in item and total scores. The DRF statistics are given in the raw score metric and, in the present case, ranged between –1 and 1 for cDIF (because each item was dichotomously scored with 1 indicating a correct response) or –26 and 26 (because the largest possible test score was 26) for cDTF, respectively. Negative values indicate that the reference group receives, on average, lower item or test scores than the comparison group, despite holding the latent proficiency in both groups comparable. In contrast, positive values indicate higher scores in the reference group. Next to the biases in the raw score metric, we also report the percentage biases cDIF% and cDTF% (Chalmers et al., 2016) that reflect the relative increase in item or test scores for the comparison group (as compared with a reference group). Finally, DRF was evaluated for the entire sample and also across specific regions of the latent variable to examine whether the assessment conditions had more pronounced effects, for example, among low-proficient children. Item parameter uncertainty was acknowledged in these analyses by repeating the DRF analyses 1,000 times for different item parameters that were randomly drawn from the asymptotic variance-covariance matrix of the parameter estimates (see Chalmers, 2018). This allowed constructing confidence intervals for the cDIF and cDTF statistics and also conducting inference tests examining the null hypothesis of no DRF.

#### Propensity Score Weighting

Because the study did not employ a true experimental design with a randomized assignment to the four assessment conditions, the different groups varied along several dimensions (see Table 1). To account for preexisting differences between children, the groups were balanced on five background characteristics (i.e., sex, age, home language, school months, and region in Germany) by estimating propensity score weights (Imai & Ratkovic, 2014). These weights were used to examine unbiased mode and settings effects for the four assessment conditions (see Kim & Walker, 2021, for a similar approach). Details on the weight estimation are summarized in the supplemental material.

### Statistical Software

The analyses were conducted in *R* version 4.1.2 (R Core Team, 2021). For the item response models and DRT analyses, we used *mirt* version 1.36.1 (Chalmers, 2012). The propensity score weights were created with *CBPS* version 0.23 (Fong et al., 2022) and *WeightIt* version 0.12.0 (Greifer, 2021).

### Transparency and Openness

For the remote assessment, the study material, detailed information on the testing procedure, and the scored reading comprehension data are available to the research community at NEPS Network (2022). Because the on-site data cannot be shared publicly due to legal restrictions, we also provide a synthetic dataset created with *synthpop* version 1.7-0 (Nowok et al., 2016) at https://osf.io/qp6gk that allows reproducing our analyses. The repository also includes the computer code and the analysis output for the reported findings.

## Results

### Description of Measurement Model

The item response model provided a satisfactory fit in each assessment condition. As expected, items in the medium third of the tests were most appropriate for the sample, as indicated by item difficulty parameters covering a range from –1.42 to 3.12 (*Mdn* = 0.52). Because the test was designed for children attending first to seventh grades, thus, covering a rather broad proficiency range, the items at the beginning of the test were rather easy for the current sample, while items located at the end of the test were rather difficult. Detailed results on the estimated item parameters and model fit are summarized in the supplemental material. We used five items with comparable difficulty parameters across the four assessment groups to place the different measurements on a common scale. Accordingly, a multi-group model with invariance constraints on the item difficulties for these five items and no constraints on the remaining items (Akaike information criterion [AIC] = 29,626, Bayesian information criterion [BIC] = 30,142) fitted comparably as a fully unrestricted model (AIC = 29640, BIC = 30220), χ^2^(12) = 10.37, *p* = .583, thus, corroborating the adopted invariance constraints.

The linked multi-group model with constraints on the anchor items fitted significantly better as compared with a model with invariance constraints on all items (AIC = 29850, BIC = 30027), χ^2^(63) = 350.00, *p* < .001. This indicates that the different assessment conditions affected the item parameters and, to some degree, did so differently for the studied items. On average, the item difficulties were slightly smaller for the paper-based test as compared with the on-site computer, Cohen’s *d* = –0.30, or the remote tablet and laptop conditions, Cohen’s *d*s = –0.10 and –0.22. In contrast, the on-site computer assessment exhibited somewhat larger difficulties than the two remote conditions at Cohen’s *d* = 0.21 and 0.09. Because differences between the testing groups were, to some extent, item-specific, the correlations of the difficulty parameters between groups can inform about the size of the heterogeneity. However, the respective correlations were rather large and fell around .98 for all comparisons. This indicates that, although item-specific differences existed, they were likely to be rather small.

### Mode and Setting Effects in Reading Comprehension

DIF was examined by calculating the pairwise differences in the item characteristics curves between the different assessment groups. The respective cDIF statistics that reflect the condition-specific biases in item scores are summarized in Table 2. A cDIF of 0 indicates no item bias, whereas negative values indicate lower item scores, on average, in the reference group (first row) as compared with the comparison group (second row), despite holding the latent proficiency in both groups constant. These results highlight significant (*p* < .05) item biases for several items. However, most effects were small and, thus, likely of negligible importance. The most pronounced effects were observed for items in the initial third of the test for which assessment modes and settings generated some cDIF. For example, for Item 6 remote settings led to item scores that were, on average, about 0.18 and 0.28 points larger as compared with on-site computer testing. In contrast, mode effects resulted in smaller expected item scores of –0.21 for the on-site computer and paper comparison. These results suggest that remote settings result in slightly higher item scores as compared with on-site assessments while mode effects reflect higher scores in paper- as compared with computer-based testing. Importantly, these effects were item-specific and, to a varying degree, limited to a few items in the first third of the test.

**Table 2.** Differential Item Functioning Statistics.

Item	Remote tablet			Remote laptop		On-site computer
	Remote laptop	On-site computer	On-site paper	On-site computer	On-site paper	On-site paper
1	0.01	0.06*	0.01	0.04	0.01	−0.05
2	0.04	0.08*	−0.14*	0.04	−0.18*	−0.23*
3	0.06	0.15*	−0.02	0.09*	−0.08	−0.18*
4	0.01	0.09*	0.04	0.07	0.03	−0.06
5	0.05	0.24*	−0.05	0.19*	−0.10*	−0.30*
6	0.11*	0.28*	0.09*	0.18*	−0.02	−0.21*
7	0.07	0.10*	−0.08*	0.03	−0.16*	−0.19*
8	−0.03	0.14*	0.07	0.17*	0.10	−0.07
9	0.03	0.13*	0.02	0.11*	0.00	−0.11*
10	0.04	−0.01	−0.04	−0.05	−0.09	−0.03
11	0.08*	0.07	0.08	−0.02	0.00	0.02
12	0.08	0.08*	−0.04	0.00	−0.11*	−0.12*
14	0.06	0.08*	0.12*	0.03	0.06	0.04
15	0.03	−0.03	0.04	−0.06	0.01	0.06
17	−0.02	−0.08*	−0.02	−0.06	0.00	0.05
18	−0.03	−0.07*	−0.06	−0.04	−0.02	0.01
19	−0.02	−0.07*	−0.02	−0.10*	−0.04	0.05
20	−0.02	−0.02	−0.02	0.00	0.00	0.00
21	−0.02	−0.06*	0.00	−0.04	0.03	0.05*
23	−0.03	−0.04*	−0.01	−0.05*	−0.01	−0.01
26	−0.04	−0.02	0.00	−0.01	0.01	−0.01

*Note*. Item bias in raw point metric (cDIF; Chalmers, 2018) with a theoretical range of −1 and 1. Positive values indicate higher expected item scores in the group in the first row as compared with the group in the second row while holding the latent proficiency constant. Items 13, 16, 22, 24, and 25 were used as anchors (see supplemental material) and, thus, are not included in the table.

* *p* < .05.

The cumulated cDIF effects across all items are reflected in the respective differential test functioning statistic cDTF, which are given in Table 3. Again, a cDTF of 0 indicates no test bias, whereas negative values indicate lower expected test scores, on average, in the reference group (first column) as compared with the comparison group (second column), despite holding the latent proficiency in both groups constant. The respective results highlighted no significant (*p* > .05) test bias within the remote setting, cDTF = 0.49, 95% CI [–0.19, 1.10], and, thus, showed no device effects. In contrast, we observed significant mode and setting effects. The remote tablet assessment led to expected test scores that were, on average, about 0.95 points, 95% CI [0.26, 1.57], higher as compared with the on-site computer condition, despite comparable proficiency distributions in both groups. However, this effect translated to a percentage bias of only about 3.65%; thus, the test scores were overestimated by less than 4%. Moreover, for the remote laptop test, the setting effect was even smaller and not significant. In contrast, the comparison of the on-site computer versus paper conditions highlighted a mode effect, cDTF = –0.90, 95% CI [–1.70, 0.16], reflecting higher expected scores for paper-based tests. Again, this translated into a rather small percentage bias corresponding to test scores overestimated by only about 3.46%.

**Table 3.** Differential Test Functioning Statistics by Latent Proficiency.

Comparison groups	Proficiency range			
	[−3, 3]	[−3, –1]	[−1, 1]	[1, 3]
Remote tablet				
Remote laptop	0.49 (1.90%)	0.79* (3.03%)	0.62 (2.40%)	0.07 (0.26%)
On-site computer	0.95* (3.65%)	1.93* (7.43%)	1.33* (5.13%)	−0.41 (−1.59%)
On-site paper	−0.04 (−0.17%)	−0.22 (−0.84%)	0.14 (0.54%)	−0.05 (−0.19%)
Remote laptop				
On-site computer	0.36 (1.37%)	1.17* (4.51%)	0.51 (1.96%)	0.61 (2.35%)
On-site paper	−0.45 (−1.72%)	−0.91 (−3.51%)	−0.36 (−1.38%)	−0.07 (−0.29%)
On-site computer				
On-site paper	−0.90* (−3.46%)	−2.12* (−8.17%)	−1.02* (−3.92%)	0.45 (1.73%)

*Note*. Test bias in raw point metric (cDTF; Chalmers, 2018) with a theoretical range of −26 and 26. The percentage bias (cDTF%) is given in the parentheses. Positive values indicate higher expected test scores in the left group as compared with the group in the second column while holding the latent proficiency constant.

* *p* < .05.

**Figure 1.**
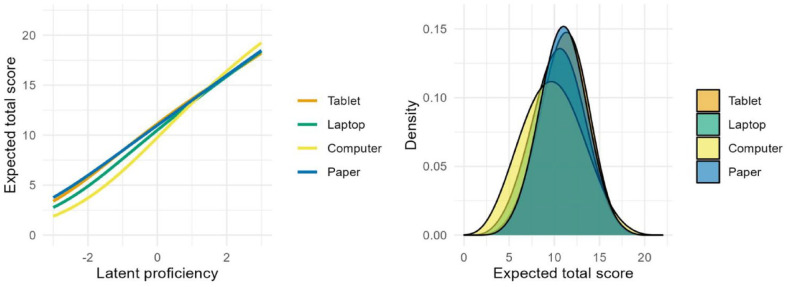
Test Scoring Functions for Assessment Condition.

To examine whether DTF varied for different levels of the latent proficiency, we also calculated these indices for low, medium, and high reading competencies (see Table 3). These analyses showed that the mode and setting effects were more pronounced at lower proficiencies, whereas for higher proficiencies the different administration conditions had no effect. For example, at low proficiency levels, remote tablet and laptop assessments showed significantly higher expected test scores as compared with the on-site computer assessments that corresponded to percentage biases of about 6.43% or 4.51%. In contrast, at high proficiencies, the respective effects were substantially smaller and not significantly different from zero. Similarly, the mode effect corresponded to a percentage bias of about 7.17% at lower proficiency and 1.73% at higher proficiencies. This interaction effect is also visualized in Figure 1 as the respective test characteristic curves for the four assessment groups (left panel), which show the expected test scores depending on the latent proficiency. These highlight notable differences between the curves at lower proficiencies with the on-site computer condition yielding lower expected total scores conditional on the same proficiency. Consequently, these differences result in slightly different test score distributions (right panel) for the four assessment conditions, although the latent proficiency is identical in all groups.

### Mode and Setting Effects in Response Effort

The average number of responses was used as an indicator of response effort. An analysis of covariance (ANCOVA) that controlled for the children’s reading comprehension, *F*(1, 1585) = 1,353.55, *p* < . 001, η^2^ = .44, found significant differences in response effort between the four groups, *F*(3, 1,585) = 17.15, *p* < . 001, η^2^ = .02. These reflected primarily mode effects because children in the on-site paper-based condition attempted fewer items as compared with the computer condition, Cohen’s *d* = 0.50 (see Table 4). The respective pairwise differences for setting effects revealed Cohen’s *d*s of –0.32 indicating lower effort in the remote conditions as compared with the on-site computer condition (with all *p*s < .001).

**Table 4.** Mean Effort and Error Score Differences between Assessment Groups.

		*M*	*SD*	Cohen’s *d* (with 95% CI)		
				1	2	3
Effort scores						
1	Remote tablet	13.58	5.28	0.00 [−0.11, 0.11]	−0.32* [−0.43, −0.21]	0.18* [0.07, 0.29]
2	Remote laptop	14.25	5.71		−0.32* [−0.46, −0.17]	0.18* [0.04, 0.33]
3	On-site computer	15.70	5.46			0.50* [0.36, 0.64]
4	On-site paper	11.31	5.11			
Error scores						
1	Remote tablet	0.17	0.20	−0.10 [−0.24, 0.03]	−0.65* [−0.78, −0.52]	0.10 [0.03, 0.23]
2	Remote laptop	0.17	0.20		−0.55* [−0.72, −0.37]	0.20* [0.03, 0.38]
3	On-site computer	0.32	0.24			0.75* [0.58, 0.92]
4	On-site paper	0.18	0.22			

*Note*. Reported are partial effect sizes controlling for reading comprehension. Positive values indicate higher conditional means in the group indicated by the row. CI = Confidence interval.

* *p* < .05.

An ANCOVA for the error rates, that is, the percentage of incorrect responses in relation to all valid responses, controlling for the children’s reading comprehension, *F*(1, 1,585) = 439.30, *p* < . 001, η^2^ = .21, also found significant differences between the four groups, *F*(3, 1585) = 34.41, *p* < . 001, η^2^ = .05. Children working in classrooms on the computer produced significantly (*p* < .05) more errors as compared with those using a paper-based test or children in the remote settings (see Table 4).

## Discussion

When psychological assessments are implemented under novel conditions, it is important to evaluate to what degree these adapted test procedures might affect the respective measurements. Otherwise, the tests might capture slightly different constructs with unknown validity and, thus, distort substantive conclusions based on them (see Flake & Fried, 2020; Schroeders & Gnambs, 2020). The recent years registered pronounced changes in the way many cognitive tests are administered. Besides a switch to computerized testing formats, these assessments were often conducted in less standardized settings, such as the test-takers’ private homes. Therefore, mode and settings effects might bias the measured constructs. The present study added to the growing field of cognitive remote testing by examining DRF in a validated test of German reading comprehension for 8-year-old children. In contrast to most previous research, the quasi-experimental design allowed us to disentangle mode from setting effects and study how each factor contributed uniquely to potential measurement biases. These analyses led to three main conclusions.

First, the move from paper- to computer-based administration resulted in mode effects, albeit to some degree differently for each item (for similar results, see Buerger et al., 2019, and Kroehne, Buerger, et al., 2019). The administration mode affected less than a quarter of all administered items and, on average, made the items more difficult for children when presented on a computer. Consequently, these item-specific differences translated into systematic biases in test scores resulting in higher expected scores for paper-based administrations. A reason for the poorer reading performance on computers might be that computer-based testing is still rather unusual in primary schools and, therefore, children are not yet accustomed to this assessment format. Unfamiliarity with computerized testing might have placed additional cognitive demands on the children that resulted in a shallower processing of the actual item content and more random guessing (see Karay et al., 2015; Leeson, 2006) which in turn led to an inferior performance on the computer-based test. Indirect support for this assumption is given in the present study by children responding to more items on the computer, but, at the same time, also producing a larger share of incorrect responses. Among the computer conditions, working on tablets most strongly resembled the paper condition, indicating that tablets might be the preferable medium in the adaption of paper-based tests in a digital format.

Second, despite implementing a proctored form of remote testing that monitored children’s testing taking by trained supervisors, small setting effects led to higher expected scores in the remote setting. Again, these setting effects were item-specific and affected only about a quarter of all administered items. An obvious speculation might be that the type of proctoring implemented in the remote setting was insufficient and some children had inadmissible support (e.g., by parents) that led to higher test scores. Although this might have contributed to the observed results to some degree, it is unlikely the only explanation. In a recent study among university students (Zinn et al., 2021), settings effects for *un*proctored remote testing were even smaller than the effect observed in the current study. Thus, it could be the case that the individual setting might have played some role because, particularly for complex tasks, the presence of others might impair performance (i.e., the *social facilitation* phenomenon; Zajonc, 1965). In support of this assumption, a meta-analytic review of studies contrasting individual versus group administrations of intelligence tests showed slightly larger task performance when no other test-takers were present (Becker et al., 2017). On a positive note, the present study found no evidence for device effects in the remote condition. Thus, the input device used to respond to the test had a negligible impact on test results.

Third, a consistent finding was that mode and settings effects did not affect all children comparably. Rather, the size of the observed differences was contingent on their latent proficiency. While children with higher reading abilities were hardly affected by changes in the administration conditions, for low ability children larger measurement biases were observed. Overall, these results replicate similar patterns that have been previously found for adolescents (Wagner et al., 2022) and young adults (Zinn et al., 2021). However, it must be emphasized that all effects found in the present study were rather small. The largest bias amounted to about 8% of the maximum test score, while most biases fell considerably below 5%. Thus, it remains to be seen whether mode and setting effects represent meaningful distortions with noteworthy consequences for applied practice.

### Implications for Remote Cognitive Testing

Remote testing does not per se seem to be inferior to on-site testing and it might even have specific advantages. First, it of course enables assessments when practical circumstances like lockdowns, long travel distances, or other obstacles prevent on-site testing. Second, it can even increase the precision of the retrieved results. The discrepancies between the on-site computer- and paper-based testing were larger than the device effects in the remote testing condition. This difference might be the consequence of group-based versus individual assessment and in the individual remote testing, they largely vanished. Thus, remote testing proved to be effective and device effect questions like using a tablet or laptop seemed to be a minor aspect in comparison. We, however, think this advantage can only play out if standardized testing situations at home can be ensured. To this end, sources of interference at home (crowded rooms, noise and music stemming from other media, interactions with other persons during the test situation) must be controlled or avoided, for example, by placing the testee in a separate room. We as well would rather prefer a proctored test delivery, as was the case in our study. Depending on the importance of the test results, especially in the case of high-stakes testing, it is important to implement measures that prevent cheating.

### Limitations and Outlook

Several weaknesses might limit the generalizability of the presented findings. First, similar to previous research (e.g., Cherry et al., 2021; Hurtz & Weiner, 2022), we did not employ a true experimental design that randomly assigned children to different administration settings. Rather, we created comparable groups using propensity score matching that has been shown to allow for meaningful analyses of mode effects (Kim & Walker, 2021). However, if systematic differences between groups remained unaccounted for, these might have distorted the reported results to some degree. For example, we cannot rule out that cohort effects might have distorted the identified setting effects to some degree because the remote test was administered at the beginning of the Corona pandemic, whereas on-site testing was conducted earlier. Therefore, future research is encouraged to replicate these findings with stronger experimental rigor. Second, administration settings are, by definition, rather heterogeneous and vary along different dimensions. For example, settings might differ with regard to the test location and the presence of others. Because the current study implemented the on-site assessment in small groups at school, we were unable to separate the two factors. It is also conceivable that difficulties in creating orderly testing conditions in the computer labs at school that was still a rather unconventional assessment approach in most primary school led to more distractions in the on-site computer condition and, thus, contributed somewhat to the observed mode effects. To identify further characteristics of the test setting that might affect performance, more specific experimental comparisons need to be implemented. Third, in the present study, the remote test was supervised by phone and a dashboard indicating the current page of the test. Although more comprehensive supervision could be achieved by video sharing that allows thoroughly monitoring of the entire test-taking conditions, this has substantially higher technological requirements (e.g., webcam, quality of the internet connection). In practice, it needs to be balanced whether the increase in control outweighs systematically excluding certain groups that do not meet the necessary computer requirements. Finally, the present analyses were limited to the measurement properties of the administered reading comprehension test. Future research should extend these findings to indicators of validity to examine whether different administration modes and settings might distort, for example, the prediction of relevant outcomes, such as school grades. Recent research also suggested that testing conditions might shape the perceptions of test-takers (Gnambs, 2022). Despite comparable test performance, test-takers rated the face validity and measurement quality of a remote test as substantially inferior to comparable on-site tests.

## Conclusion

Taken together, in remote testing situations, the data collection can be as precise as in on-site testing and consequently, we can encourage more progressive use of this assessment format. At the same time, mode effects in touch screen delivered assessment are quite small in comparison to paper-based testing over the complete range of the latent ability. Consequently, using tablets might mitigate discrepancies between paper-based and digitally delivered testing situations.

## Supplemental Material

sj-docx-1-asm-10.1177_10731911231159369 – Supplemental material for Remote Testing of Reading Comprehension in 8-Year-Old Children: Mode and Setting Effects Click here for additional data file. Supplemental material, sj-docx-1-asm-10.1177_10731911231159369 for Remote Testing of Reading Comprehension in 8-Year-Old Children: Mode and Setting Effects by Timo Gnambs and Wolfgang Lenhard in Assessment

## Notes

1. The school month does not refer to the month of the year, but the number of months since the beginning of the current school year (see Lenhard, Lenhard, & Schneider, 2017). Because the beginning of the school year slightly differs between the German federal states, the same school month might refer to different months of the year.

2. Mode effects for the full norm sample including first to sixth graders haven been previously reported in the work of Lenhard, Schroeders, and Lenhard (2017). However, this did not include detailed differential response functioning analyses as presented in the current paper.
