# Supplementary material for: Remote Testing of Reading Comprehension in 8-Year-Old Children: Mode and Setting Effects
Source: Assessment. 2023 Mar 8;31(2):248–62. doi: 10.1177/10731911231159369 (PMC10822056; doi:10.1177/10731911231159369)
Supplement: sj-docx-1-asm-10.1177_10731911231159369 – Supplemental material for Remote Testing of Reading Comprehension in 8-Year-Old Children: Mode and Setting Effects [file sj-docx-1-asm-10.1177_10731911231159369.docx]

Supplemental Material for

**Remote Testing of Reading Comprehension in 8-Year-Old Children:
Mode and Setting Effects**

Propensity Score Weighting 2

Item Response Modeling 5

Identification of Anchor Items 11

Sensitivity Analyses 13

Additional References 15

# Propensity Score Weighting

The assessment conditions varied along with several characteristics because children were not randomly assigned to the different groups. While the remote samples were assessed in 2020 as part of the NEPS (Blossfeld & Roßbach, 2019), the on-site assessments were part of the norm sample of the ELFE-II (Lenhard et al., 2017a) that was collected in 2015. Moreover, the two remote conditions for children working on a tablet or a laptop were determined by the available computer device in the household. Tablets were the recommended choice, whereas laptops served as a replacement if no laptop was available. Because of the non-random assignment of children to the different assessment groups, preexisting differences between children might confound group comparison. The descriptive sample statistics in Table 1 show that, for example, the remote samples included fewer children with a home language other than German (19%) as compared to the on-site samples (30%). Moreover, the latter was tested about one month earlier in the school year because in contrast to the remote assessments testing did not take place during the school holidays.

**Table S1**

*Summary of Propensity Score Weights by Assessment Group*

|  | *N* | *Mdn* | IQR | *Max* |
| --- | --- | --- | --- | --- |
| Remote sample: tablet | 998 | 0.96 | [0.83, 1.12] | 2.72 |
| Remote sample: laptop | 185 | 0.95 | [0.81, 1.11] | 2.53 |
| On-site sample: computer | 200 | 0.70 | [0.43, 1.29] | 3.00 |
| On-site sample: paper | 207 | 0.53 | [0.43, 1.24] | 3.00 |

*Note*. IQR = interquartile range.

Therefore, the four assessment groups (i.e., remote tablet, remote laptop, on-site computer, on-site paper) were balanced on five background characteristics including the children’s sex (coded 1 for girls and 0 for boys), age (in years), home language (coded 0 for German and 1 for non-German), school months, and geographical region (coded 1 for North Germany and 0 for South Germany). We estimated covariate balancing propensity score weights (Imai & Ratkovic, 2014) using the *R* package *CBPS* version 0.22 (Fong, Ratkovic, & Imai, 2021). Generally, weights should exhibit a rather small spread (Théberge, 2000). Otherwise, weighted estimates can be unduly influenced by a few observations with very large weights, while observations with rather small weights hardly contribute to the analyses. Therefore, we employed iterative trimming to avoid extreme weights (cf. Kolenikov, 2014) and generated weights ranging from 0.33 to 3.00. The resulting weights are summarized in Table S1.

**Figure S1**

*Covariance Balance across Assessment Groups*


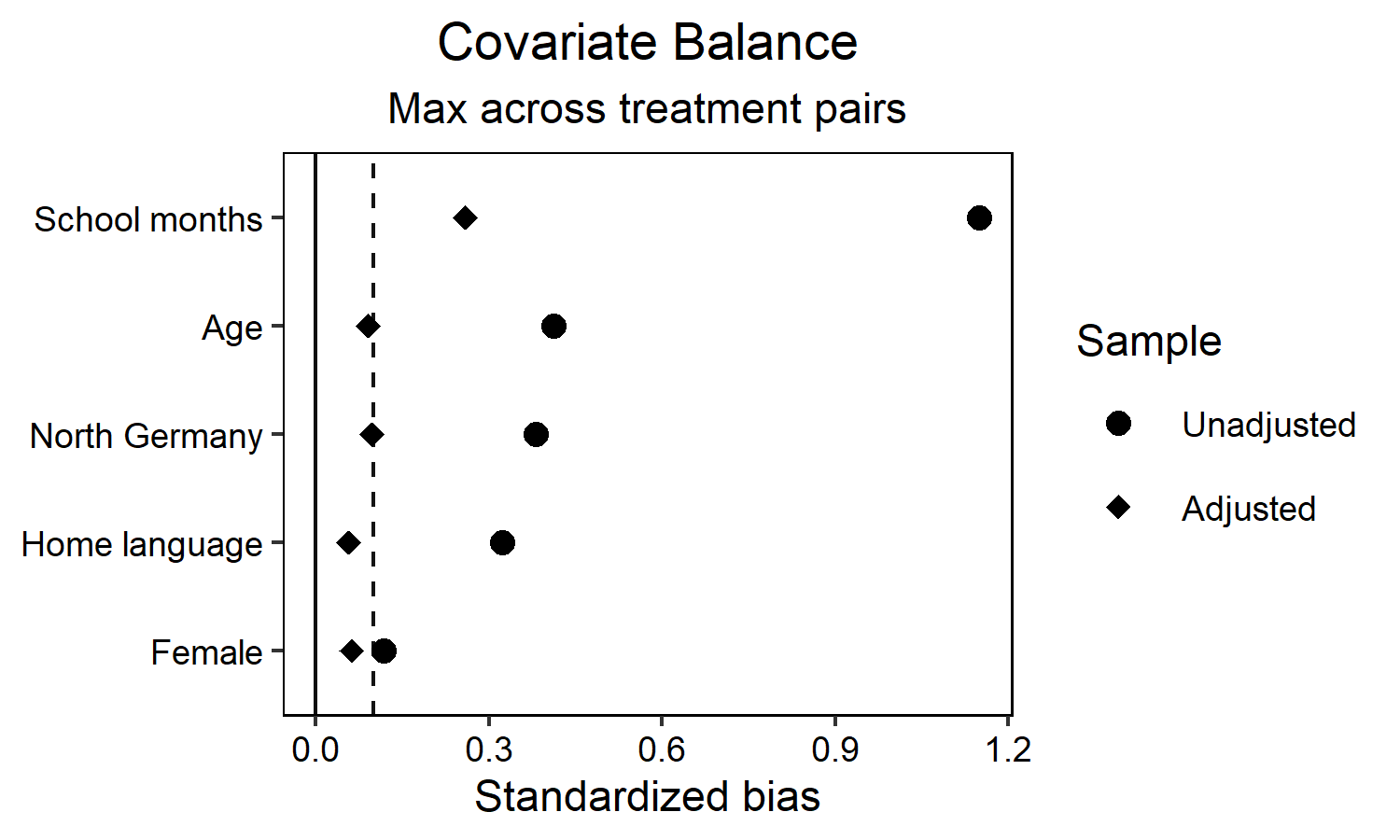


Figure S1 presents the standardized bias (i.e., standardized mean differences for continuous covariates and standardized difference in proportions for binary covariates) before (circles) and after (diamonds) weighting. Whereas the four groups were highly unbalanced regarding school months and, to a lesser degree, also regarding age, home language, and geographical region, the sex distribution was roughly comparable across groups. Applying the estimated propensity score weights, resulting in a substantial reduction in bias that fell below 0.10 (dashed lined) for sex, age, home language, and the geographical region. Although the school months were also substantially better balanced after weighting, a slight bias of *d* = 0.26 remained. However, the observed school months had a severely restricted range in the examined samples falling between 9 and 13. Therefore, the standardized effect size does not properly reflect practically meaningful differences. The consequences of the covariate balancing for the sample characteristics are shown in Table 1. The weighted samples showed considerable improvements in the balance of the covariates and, thus, created highly comparable groups. Importantly, the remaining standardized bias in the school months translated to a difference of about 0.22 months between the remote and on-site samples which were considered negligible.

# Item Response Modeling

Preliminary analyses evaluated the fit of the one-parametric item response model (Rasch, 1960) that underlies the scoring rationale of the test (see Lenhard et al., 2017a) in the four assessment conditions. In the first step, four different models were fitted to the data: (a) a one-parametric logistic model (1PL; Rasch, 1960) that only considered different item difficulties but assumed equal discrimination parameters of 1, (b) an extended one-parametric logistic model (see Kubinger & Draxler, 2007) with guessing parameters fixed to 0.25 (i.e., reflecting the guessing probability of a multiple-choice item with one correct and three incorrect response options), (c) a two-parametric logistic model with constrained discrimination parameters that assumed identical discriminations across items, and (d) an unconstrained two-parametric logistic model with item-specific discrimination parameters (2PL; Birnbaum, 1968). These models were compared using likelihood ratio tests and information criteria. Because the two one-parametric models estimate the same number of parameters, respective model comparisons are limited to information criteria.

The model comparisons in Table S2 showed that the 1PL and constrained 2PL exhibited comparable fits in all four conditions, while the unconstrained 2PL fitted better to the data. However, an inspection of the item parameters of the 2PL showed that several items exhibited extreme difficulty parameters exceeding $\pm10$ and very low discrimination parameters falling below .50. Moreover, acknowledging guessing did not improve model fit. Therefore, despite the empirical preference for the more complex 2PL, we adopted the 1PL because it more closely aligned with the theoretical construction rationale of the test (Lenhard et al., 2017a). However, sensitivity analyses (see below) evaluated the robustness of our results and replicated the DRF analyses using the 2PL.

**Table S2**

*Model Comparisons by Assessment Conditions*

|  | *logLik* | Parameters | AIC | BIC | Δχ^2^ | *df* | *p* |
| --- | --- | --- | --- | --- | --- | --- | --- |
| Remote sample: tablet |  |  |  |  |  |  |  |
| Unconstrained 1PL | -8977 | 27 | 18007 | 18139 |  |  |  |
| Extended 1PL | -11060 | 27 | 22174 | 22307 |  |  |  |
| Constrained 2PL | -8976 | 28 | 18008 | 18145 | 1 | 1 | .336 |
| Unconstrained 2PL | -8495 | 52 | 17094 | 17349 | 962 | 24 | < .001 |
| Remote sample: laptop |  |  |  |  |  |  |  |
| Unconstrained 1PL | -1765 | 27 | 3585 | 3672 |  |  |  |
| Extended 1PL | -2085 | 27 | 4224 | 4311 |  |  |  |
| Constrained 2PL | -1765 | 28 | 3586 | 3676 | 1 | 1 | .431 |
| Unconstrained 2PL | -1679 | 52 | 3461 | 3629 | 173 | 24 | < .001 |
| On-site sample: computer |  |  |  |  |  |  |  |
| Unconstrained 1PL | -2194 | 27 | 4442 | 4531 |  |  |  |
| Extended 1PL | -2459 | 27 | 4971 | 5061 |  |  |  |
| Constrained 2PL | -2194 | 28 | 4444 | 4536 | 1 | 1 | .997 |
| Unconstrained 2PL | -2113 | 52 | 4329 | 4501 | 163 | 24 | < .001 |
| On-site sample: paper |  |  |  |  |  |  |  |
| Unconstrained 1PL | -1776 | 27 | 3606 | 3696 |  |  |  |
| Extended 1PL | -2276 | 27 | 4606 | 4697 |  |  |  |
| Constrained 2PL | -1776 | 28 | 3608 | 3701 | 1 | 1 | .430 |
| Unconstrained 2PL | -1684 | 52 | 3472 | 3645 | 184 | 24 | < .001 |

*Note*. 1PL = One-parametric logistic test model (Rasch, 1960); Extended 1PL = 1PL with fixed guessing parameter (Kubinger & Draxler, 2007); 2PL = Two-parametric logistic test model (Birnbaum, 1968); Δχ^2^ = Log-likelihood ratio test comparing a given model with the previous more restrictive model; AIC = Akaike’s information criterion; BIC = Bayesian information criterion.

As expected, not all items were equally informative for the studied samples (see Tables S3 and S4). Because the administered test was constructed for children in Grades 1 to 7, the first items were rather easy for the homogenous age group examined in the present study, while the last items were rather difficult. Consequently, primarily items with medium difficulty that matched the proficiency distribution of the current samples were most informative for the latent proficiency estimation. The Wright Maps in Figure S2 show the latent proficiency distribution on the left and the item difficulties in the four conditions on the right. These highlight that in each sample the items covered a broad range to precisely measure the children’s text comprehension abilities.

**Table S3**

*Item Parameters and Item Fits for Remote Assessments*

|  | Remote tablet | | | | Remote laptop | | |  |
| --- | --- | --- | --- | --- | --- | --- | --- | --- |
| Item | % correct | *b* | WMNSQ (*z*) | a*Q*3 | % correct | *b* | WMNSQ (*z*) | a*Q*3 |
| 1 | 97% | -4.52 | 1.09 (0.55) | .07 | 98% | -4.62 | 1.24 (0.63) | .11 |
| 2 | 81% | -2.16 | 1.19 (3.61) | .09^***^ | 82% | -2.20 | 1.14 (1.17) | .10 |
| 3 | 85% | -2.52 | 1.09 (1.43) | .11^*^ | 84% | -2.31 | 1.08 (-0.67) | .10 |
| 4 | 86% | -2.60 | 1.03 (0.54) | .09^+^ | 88% | -2.80 | 1.04 (0.27) | .13 |
| 5 | 78% | -1.87 | 1.18 (3.90) | .09^***^ | 78% | -1.86 | 1.10 (0.99) | .11 |
| 6 | 83% | -2.29 | 1.04 (0.78) | .10^***^ | 77% | -1.80 | 1.16 (1.54) | .13 |
| 7 | 81% | -2.19 | 0.92 (-1.66) | .10 | 80% | -1.98 | 0.90 (-0.94) | .13 |
| 8 | 69% | -1.24 | 0.90 (-2.91) | .07 | 77% | -1.74 | 0.88 (-1.25) | .08 |
| 9 | 65% | -0.98 | 0.84 (-4.79) | .08^*^ | 68% | -1.15 | 0.83 (-2.34) | .10 |
| 10 | 68% | -1.18 | 0.72 (-8.56) | .09^***^ | 69% | -1.23 | 0.78 (-2.97) | .11 |
| 11 | 52% | -0.18 | 0.84 (-4.91) | .09^***^ | 49% | -0.03 | 0.94 (-0.72) | .10 |
| 12 | 50% | -0.05 | 0.72 (-8.73) | .09^***^ | 47% | 0.07 | 0.76 (-3.28) | .07 |
| 13 | 43% | 0.37 | 0.63 (11.21) | .12^***^ | 46% | 0.11 | 0.66 (-4.79) | .12 |
| 14 | 38% | 0.67 | 0.68 (-9.09) | .12^***^ | 37% | 0.70 | 0.70 (-3.60) | .10 |
| 15 | 32% | 1.07 | 0.68 (-8.37) | .11^***^ | 33% | 0.95 | 0.74 (-2.89) | .14 |
| 16 | 25% | 1.58 | 0.67 (-7.90) | .09^***^ | 30% | 1.14 | 0.74 (-2.67) | .12 |
| 17 | 13% | 2.75 | 0.93 (-0.97) | .06 | 18% | 2.18 | 0.99 (-0.06) | .09 |
| 18 | 12% | 2.92 | 0.75 (-3.61) | .08^***^ | 18% | 2.19 | 0.67 (-2.63) | .10 |
| 19 | 10% | 3.28 | 0.92 (-0.86) | .13^+^ | 9% | 3.27 | 0.88 (-0.53) | .08 |
| 20 | 7% | 3.70 | 0.79 (-2.15) | .12^+^ | 10% | 3.05 | 0.97 (-0.09) | .14 |
| 21 | 7% | 3.72 | 0.81 (-1.94) | .14^*^ | 11% | 2.95 | 0.84 (-0.82) | .12 |
| 22 | 5% | 4.29 | 0.84 (-1.25) | .12^+^ | 7% | 3.69 | 0.98 (-0.01) | .13 |
| 23 | 4% | 4.53 | 0.97 (-0.19) | .11^+^ | 5% | 4.26 | 0.91 (-0.21) | .14 |
| 24 | 3% | 4.87 | 0.93 (-0.42) | .11 | 3% | 5.05 | 0.90 (-0.14) | .12 |
| 25 | 2% | 5.49 | 0.85 (-0.70) | .09 | 3% | 4.86 | 0.86 (-0.26) | .13 |
| 26 | 1% | 6.13 | 1.08 (0.39) | .07 | 3% | 4.75 | 0.80 (-0.49) | .10 |

*Note*. *b* = Difficulty parameter; WMNSQ = Weighted mean square statistic (with *z*-value). a*Q*3 = Mean absolute adjusted *Q*3 statistic with Chalmers and Ng’s (2017) inference test for item misfit with Benjamini and Hochberg (1995) correction.

^***^ *p* < .001, ^**^ *p* < .01, ^*^ < .05, ^+^ *p* < .10

**Table S4**

*Item Parameters and Item Fits for On-Site Assessments*

|  | On-site computer | | | | On-site paper | | | |
| --- | --- | --- | --- | --- | --- | --- | --- | --- |
| Item | % correct | *b* | WMNSQ (*z*) | a*Q*3 | % correct | *b* | WMNSQ (*z*) | a*Q*3 |
| 1 | 95% | -3.40 | 0.57 (-1.78) | .05 | 91% | -3.51 | 1.07 (0.44) | .08 |
| 2 | 80% | -1.73 | 1.00 (0.07) | .09 | 88% | -3.15 | 1.19 (1.78) | .05 |
| 3 | 77% | -1.50 | 0.89 (-1.24) | .12 | 78% | -2.06 | 0.89 (-1.03) | .05 |
| 4 | 83% | -1.98 | 1.12 (0.94) | .09 | 72% | -1.64 | 1.07 (0.82) | .09 |
| 5 | 60% | -0.53 | 1.06 (0.96) | .10 | 72% | -1.63 | 0.95 (-0.54) | .09 |
| 6 | 61% | -0.56 | 1.02 (0.32) | .15 | 63% | -1.01 | 0.97 (-0.34) | .10 |
| 7 | 79% | -1.61 | 0.93 (-0.69) | .12 | 81% | -2.39 | 0.86 (-1.33) | .07 |
| 8 | 61% | -0.59 | 0.99 (-0.14) | .13 | 51% | -0.20 | 0.82 (-2.33) | .08 |
| 9 | 57% | -0.39 | 0.89 (-2.11) | .08 | 51% | -0.23 | 0.85 (-1.91) | .10 |
| 10 | 76% | -1.42 | 0.94 (-0.67) | .09 | 61% | -0.84 | 0.85 (-2.13) | .08 |
| 11 | 49% | 0.01 | 0.85 (-2.84) | .08 | 35% | 0.91 | 0.90 (-0.98) | .09 |
| 12 | 46% | 0.20 | 0.77 (-4.34) | .09 | 43% | 0.32 | 0.79 (-2.48) | .11 |
| 13 | 45% | 0.25 | 0.79 (-3.91) | .11 | 35% | 0.91 | 0.74 (-2.69) | .13 |
| 14 | 30% | 1.01 | 0.86 (-1.74) | .11 | 21% | 2.15 | 0.67 (-2.50) | .12 |
| 15 | 36% | 0.72 | 0.81 (-2.79) | .13 | 22% | 1.96 | 0.64 (-2.95) | .13 |
| 16 | 22% | 1.53 | 0.81 (-1.90) | .12 | 19% | 2.36 | 0.51 (-3.94) | .11 |
| 17 | 19% | 1.77 | 0.91 (-0.76) | .11 | 13% | 3.12 | 0.67 (-2.11) | .11 |
| 18 | 17% | 1.95 | 0.90 (-0.76) | .11 | 14% | 2.91 | 0.53 (-3.38) | .14 |
| 19 | 15% | 2.17 | 1.06 (0.42) | .13 | 10% | 3.63 | 0.70 (-1.67) | .21 |
| 20 | 7% | 3.13 | 1.09 (0.45) | .10 | 8% | 4.03 | 0.89 (-0.48) | .18 |
| 21 | 11% | 2.59 | 0.71 (-1.75) | .11 | 6% | 4.38 | 0.90 (-0.41) | .15 |
| 22 | 6% | 3.39 | 1.31 (1.12) | .15 | 5% | 4.89 | 0.72 (-1.10) | .11 |
| 23 | 6% | 3.23 | 1.18 (0.74) | .14 | 5% | 4.89 | 0.72 (-1.12) | .13 |
| 24 | 2% | 4.68 | 0.70 (-0.44) | .10 | 4% | 5.21 | 0.93 (-0.14) | .12 |
| 25 | 2% | 4.56 | 1.25 (0.62) | .12 | 1% | 6.86 | 1.62 (1.11) | .13 |
| 26 | 2% | 4.35 | 1.32 (0.78) | .11 | 1% | 6.42 | 0.51 (-1.07) | .10 |

*Note*. *a* = Discrimination parameter, *b* = Difficulty parameter; WMNSQ = Weighted mean square statistic (with *z*-value). a*Q*3 = Mean absolute adjusted *Q*3 statistic with Chalmers and Ng’s (2017) inference test for item misfit with Benjamini and Hochberg (1995) correction.

^***^ *p* < .001, ^**^ *p* < .01, ^*^ < .05, ^+^ *p* < .10

Item fit for the 1PL was examined for each assessment condition using the weighted mean square (WMNSQ) statistic (Linacre & Wright, 1994) and the adjusted *Q*3 statistic for local independence (Yen, 1993). The WMNSQ has an expectation of 1 and indicates more variation (or noise) than predicted by the item response model (i.e., an underfit) for values greater than 1. Following prevalent practice (e.g., Smith et al., 2008), we considered values of WMNSQ < 1.15 as indicative of close item fit, 1.15 ≤ WMNSQ < 1.20 as small item misfit, and WMNSQ ≥ 1.20 as considerable item misfit. We do not rely on the associated inference test because it is strongly affected by the sample size (Karabatsos, 2000; Linacre, 2003). Rather, we adopt the more conservative test statistic proposed by Chalmers and Ng (2017) with Benjamini and Hochberg (1995) corrected *p*-values. The local independence assumption of the 1PL was evaluated by inspecting the residual correlations of the model. For each item, we report the mean absolute adjusted *Q*3 statistic (Yen, 1993) across the respective item pairs. Values less than .20 are typically considered indicative of essential unidimensionality (cf. Chen & Thissen, 1997).

The item response model provided a satisfactory fit in each assessment condition (see Table S3 and S4). Although some significant (*p* < .05) model violations were observed in the remote tablet condition, all weighted mean square statistics fell below the recommended threshold of 1.20, thus, indicating acceptable item fits for each test. Also, Yen’s (1993) adjusted *Q*_3_ statistic did not identify noteworthy local item dependencies. Together, these results highlight an appropriate unidimensional item response model in each testing condition.

**Figure S2**

*Wright Maps by Assessment Conditions*


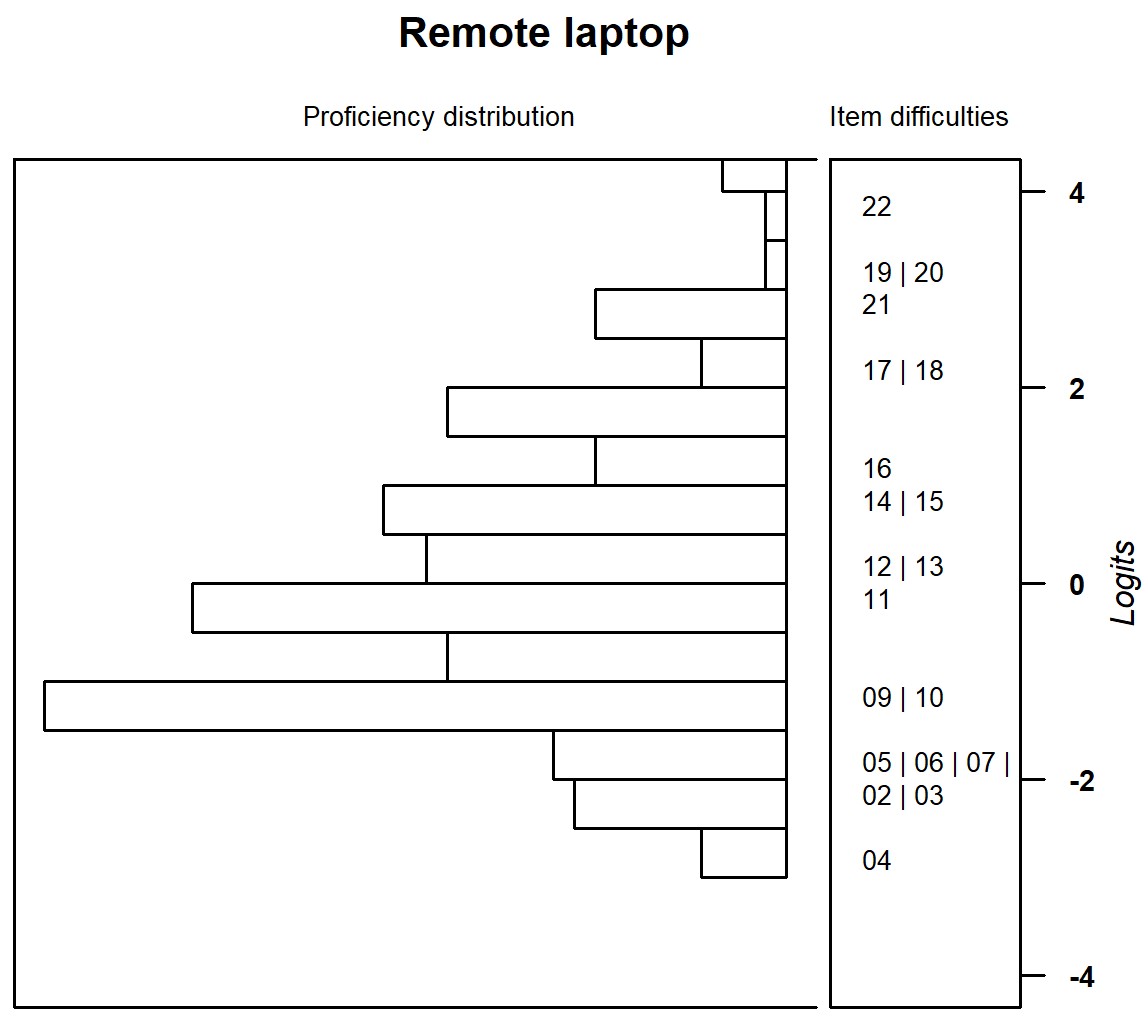

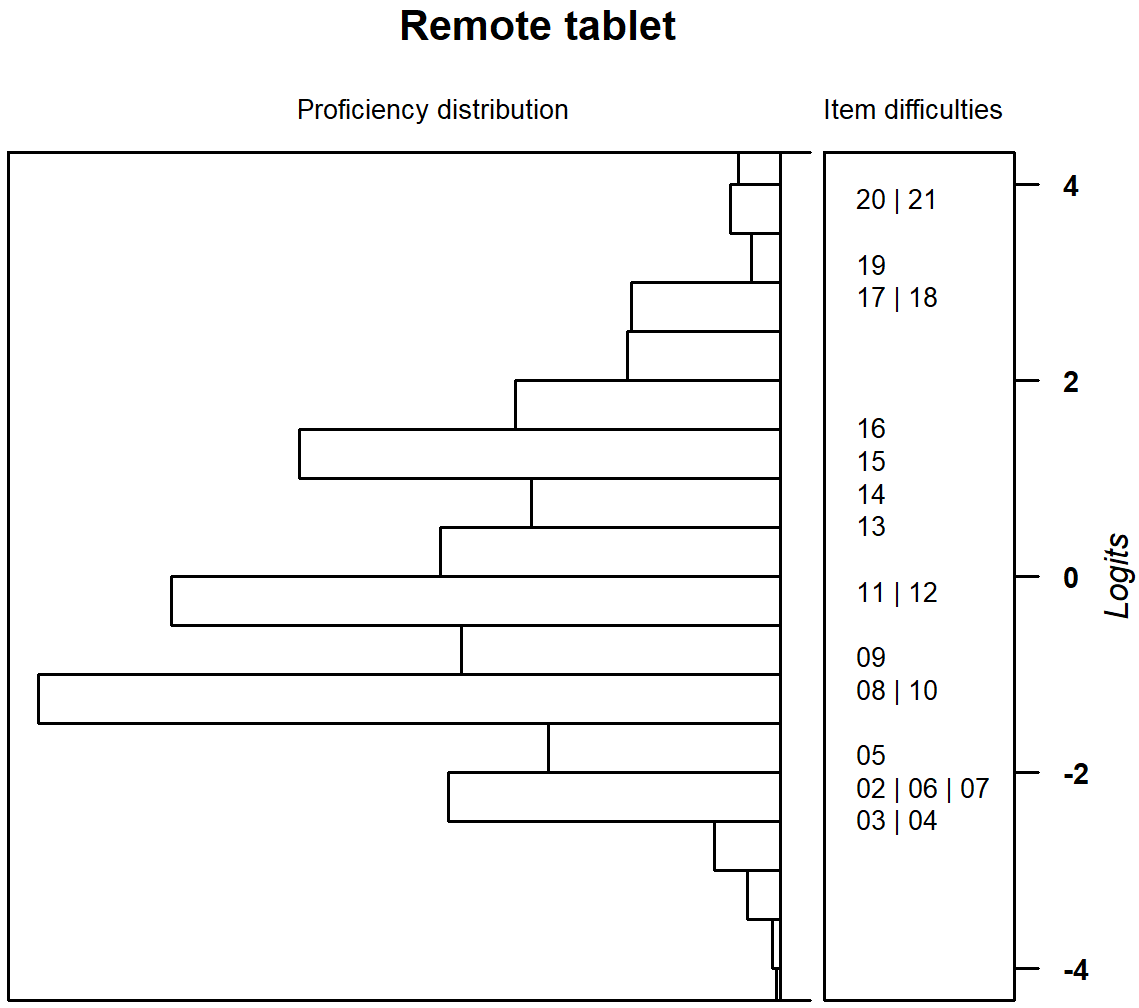


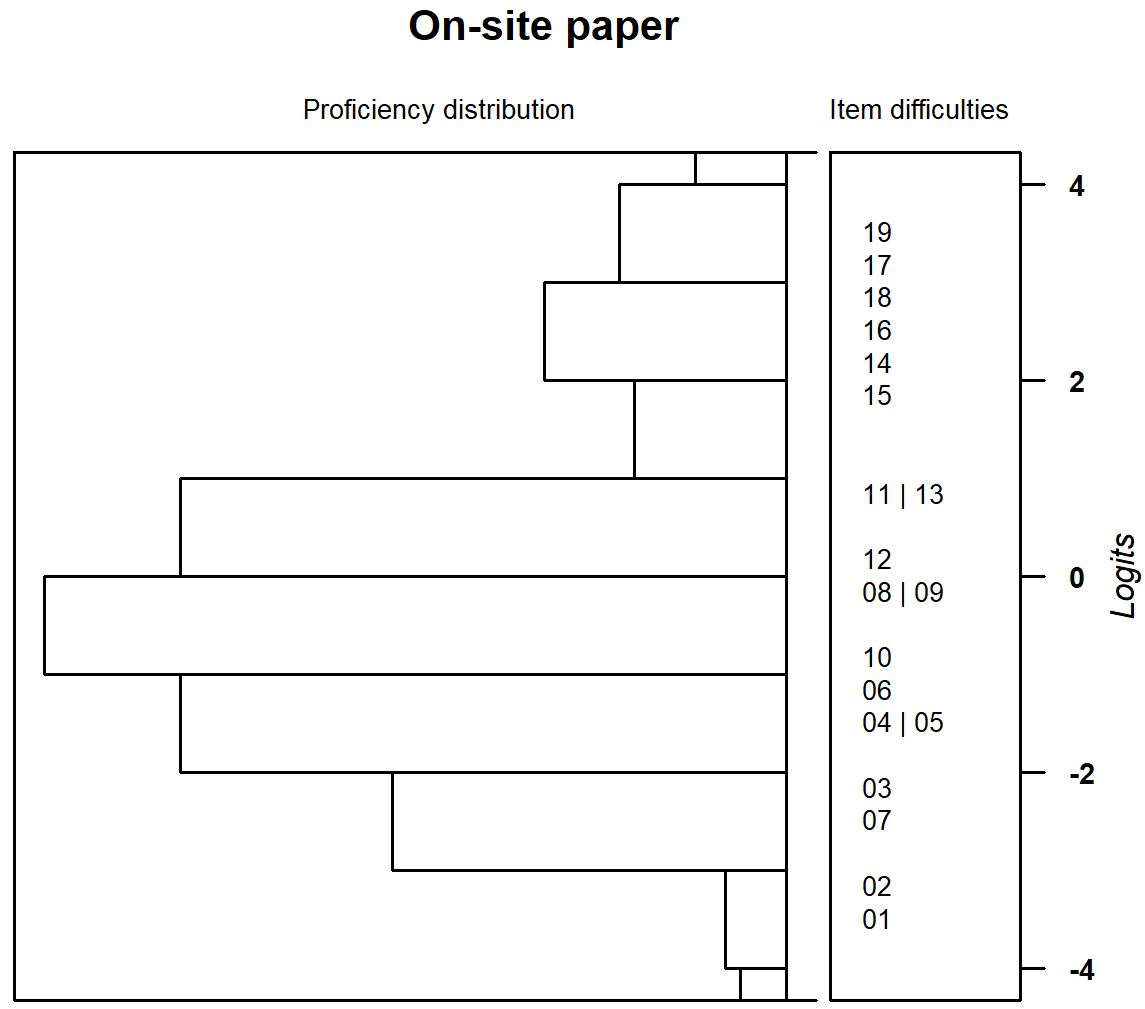

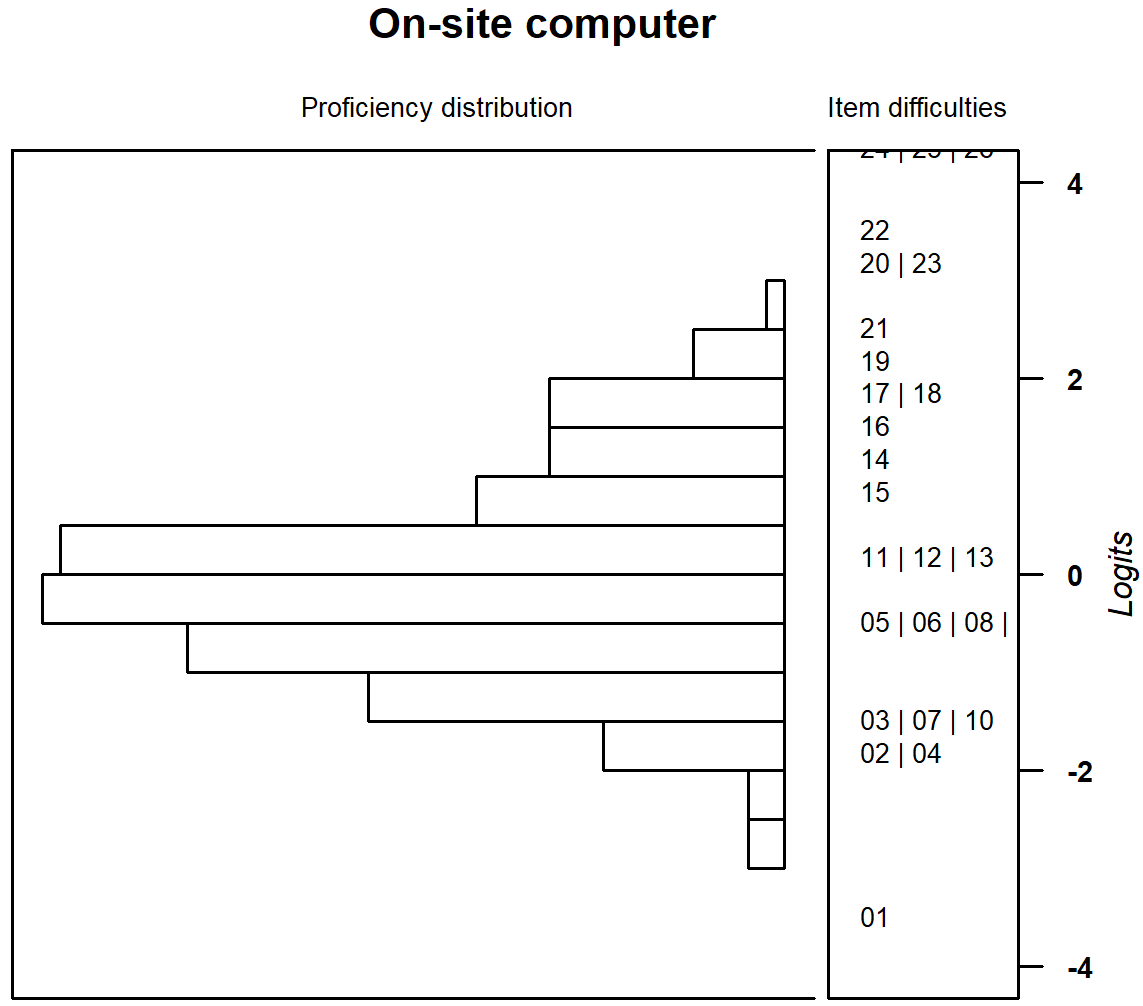


*Note*. The item numbers correspond to the numbering in Tables S3 and S4. Items with difficulties falling outside the range of -4 to +4 are not presented.

# Identification of Anchor Items

Comparisons between different groups require placing the different measurements on a common scale. To identify invariant items across the four assessment conditions, a series of multi-group item response models were estimated following Woods (2009). First, a fully constrained baseline model was estimated that constrained all item difficulties and item discriminations across groups, but freely estimated factor means and variances for the laptop, computer, and paper conditions. In the remote tablet condition, the latter were constrained to 0 and 1, respectively, for model identification. Then, a series of less restrictive models were estimated that freed the parameter constraints for one item at a time. Model comparisons between the fully restricted model and the less restricted model for each item identified anchor items with measurement invariant parameters across assessment conditions based on likelihood-ratio tests with Benjamini and Hochberg (1995) correction (cf. Woods, 2009). The respective results summarized in Table S5 identified several invariant items. For the present analyses, we selected items 13, 16, 22, 24, and 25 that exhibited the smallest χ^2^-statistics. Accordingly, the multi-group model with constraints on these five anchor items was used for the differential response functioning analyses.

**Table S5**

*Comparisons between Fully Restricted Model and Itemwise Less Restricted Models*

| Item | ΔAIC | ΔBIC | χ^2^(4) | *p* |
| --- | --- | --- | --- | --- |
| 1 | 1.94 | 18.05 | 4.06 | .368 |
| 2 | -36.91 | -20.80 | 42.10 | < .001 |
| 3 | -3.62 | 12.49 | 9.62 | .044 |
| 4 | 0.71 | 16.83 | 5.29 | .232 |
| 5 | -30.84 | -14.72 | 36.84 | < .001 |
| 6 | -53.10 | -36.98 | 59.10 | < .001 |
| 7 | -9.93 | 6.18 | 15.93 | .004 |
| 8 | -9.54 | 6.58 | 15.54 | .005 |
| 9 | 2.37 | 18.49 | 3.63 | .407 |
| 10 | -5.78 | 10.34 | 11.78 | .021 |
| 11 | -2.04 | 14.07 | 8.04 | .083 |
| 12 | 2.56 | 18.68 | 3.44 | .407 |
| 13 | 2.47 | 18.59 | 3.53 | .407 |
| 14 | -8.71 | 7.41 | 14.71 | .006 |
| 15 | -3.72 | 12.39 | 9.72 | .044 |
| 16 | 3.78 | 19.90 | 2.22 | .562 |
| 17 | -10.00 | 6.11 | 16.00 | .004 |
| 18 | -10.61 | 5.51 | 16.61 | .004 |
| 19 | -10.46 | 5.65 | 16.46 | .004 |
| 20 | 3.84 | 19.96 | 2.16 | .562 |
| 21 | -5.49 | 10.62 | 11.49 | .022 |
| 22 | 2.97 | 19.08 | 3.03 | .457 |
| 23 | -1.92 | 14.19 | 7.92 | .083 |
| 24 | 4.45 | 20.57 | 1.55 | .672 |
| 25 | 3.17 | 19.28 | 2.83 | .473 |
| 26 | -0.73 | 14.38 | 6.73 | .132 |

*Note*. ΔAIC = Difference in Akaike’s information criteria (Akaike, 1974), ΔBIC = Difference in Bayesian information criteria (Schwarz, 1978), χ^2^ = Likelihood-ratio test statistic, *p* = *p*-value for χ^2^ with Benjamini and Hochberg (1995) correction. Positive information criteria indicate a better fit for the less restricted model. Gray rows highlight selected anchor items.

# Sensitivity Analyses

Although the scoring scheme developed by the test authors (Lenhard et al, 2017a) corresponded to the one-parametric logistic test model (Rasch, 1960), the empirical analyses suggested a superior fit of the two-parametric logistic test model (Birnbaum, 1968) that also allows for different discrimination parameters. To examine to what degree the choice of the item response model affected the DRF analyses, these were repeated for the 2PL. The respective DIF and DTF results are summarized in Tables S6 and S7.

**Table S6**

*Differential Item Functioning Statistics for Two-Parametric Logistic Test Model*

|  | Remote tablet | | | Remote laptop | | On-site computer | |
| --- | --- | --- | --- | --- | --- | --- | --- |
| Item | Remote laptop | On-site computer | On-site paper | On-site computer | On-site paper | | On-site paper |
| 1 | -0.01 | 0.09^*^ | 0.06^**^ | 0.06^+^ | 0.06^*^ | | -0.01 |
| 2 | -0.01 | 0.01 | -0.08^*^ | 0.02 | -0.07^+^ | | -0.13^**^ |
| 3 | 0.02 | 0.13^**^ | 0.06^*^ | 0.09^*^ | 0.04 | | -0.09^+^ |
| 4 | -0.02 | 0.05 | 0.12^***^ | 0.06 | 0.14^***^ | | 0.06 |
| 5 | 0.00 | 0.20^***^ | 0.04 | 0.19^***^ | 0.04 | | -0.18^***^ |
| 6 | 0.06^+^ | 0.23^***^ | 0.17^***^ | 0.17^***^ | 0.11^*^ | | -0.08 |
| 7 | 0.03 | 0.10^*^ | -0.01 | 0.05 | -0.04 | | -0.14^**^ |
| 8 | -0.06 | 0.12^**^ | 0.14^***^ | 0.18^***^ | 0.21^***^ | | 0.02 |
| 9 | 0.00 | 0.14^***^ | 0.10^**^ | 0.14^**^ | 0.10^+^ | | -0.05 |
| 10 | 0.03 | 0.04 | 0.03 | 0.01 | 0.00 | | -0.01 |
| 11 | 0.07^+^ | 0.08^*^ | 0.12^**^ | 0.00 | 0.06 | | 0.04 |
| 12 | 0.07^+^ | 0.08^**^ | 0.00 | 0.01 | -0.06 | | -0.10^*^ |
| 14 | 0.06^+^ | 0.07^*^ | 0.11^***^ | 0.01 | 0.05 | | 0.05 |
| 15 | 0.03 | -0.04 | 0.01 | -0.08^+^ | -0.01 | | 0.07 |
| 17 | -0.02 | -0.09^***^ | -0.04 | -0.07^+^ | -0.01 | | 0.06^+^ |
| 18 | -0.03 | -0.09^***^ | -0.06^*^ | -0.07^+^ | -0.03 | | 0.02 |
| 19 | 0.02 | -0.08^**^ | -0.03 | -0.10^**^ | -0.05 | | 0.05^+^ |
| 20 | -0.02 | -0.01 | -0.03 | 0.00 | -0.01 | | 0.00 |
| 21 | -0.02 | -0.06^*^ | -0.01 | -0.05 | 0.01 | | 0.05^*^ |
| 23 | 0.00 | -0.03 | -0.02 | -0.03 | -0.02 | | 0.02 |
| 26 | -0.02 | -0.01 | 0.00 | 0.00 | 0.01 | | 0.01 |

*Note*. sDIF statistics in raw point metric (cf. Chalmers, 2018). Positive values indicate higher expected item scores in the group in the first row as compared to the group in the second row while holding the latent proficiency constant. Items 13, 16, 22, 24, and 25 were used as anchors (see supplemental material) and, thus, are not included in the table.

^***^ *p* < .001, ^**^ *p* < .01, ^*^ < .05, ^+^ *p* < .10

**Table S7**

*Differential Test Functioning Statistics for Two-Parametric Logistic Test Model*

| Comparison groups | | Proficiency range | | | |
| --- | --- | --- | --- | --- | --- |
|  |  | [-3, 3] | [-3, 1] | [-1, 1] | [1, 3] |
| Remote tablet |  |  |  |  |  |
|  | Remote laptop | -0.09 (-0.33%) | -0.44 (-1.69%) | 0.30 (1.16%) | -0.12 (-0.45%) |
|  | On-site computer | 0.96^***^ (3.68%) | 1.65^*^ (6.34%) | 1.03^***^ (3.96%) | 0.19 (0.74%) |
|  | On-site paper | 0.58^*^ (2.34%) | 2.37^***^ (9.11%) | 0.76^*^ (2.93%) | -1.39^*^ (-5.33%) |
| Remote laptop |  |  |  |  |  |
|  | On-site computer | 1.02^**^ (3.94%) | 2.14^*^ (8.23%) | 0.58^+^ (2.24%) | 0.35 (1.35%) |
|  | On-site paper | 0.60^+^ (2.31%) | 2.71^**^ (10.44%) | 0.29 (1.13%) | -1.20^*^ (-4.64%) |
| On-site computer |  |  |  |  |  |
|  | On-site paper | -0.49 (-3.89%) | -0.01 (-0.03%) | -0.32 (-1.22%) | -1.13 (-4.38%) |

*Note*. sDTF statistics in raw point metric with the percentage of the maximum score in parentheses (sDTF %; cf. Chalmers, 2018). Positive values indicate higher expected test scores in the left group while holding the latent proficiency constant.

^***^ *p* < .001, ^**^ *p* < .01, ^*^ < .05, ^+^ *p* < .10

The DRF analyses replicated the analyses for the 1PL presented in the main manuscript to a large part. About a third of the items exhibited significant DIF reflecting higher item scores in the remote settings and for the paper-based administrations (see Table S6). The DTF analyses showed that these translated into test score biases advantaging children taking the remote tests (Table S7). Again, this effect was more pronounced for low-ability children as compared to children with higher reading abilities. In contrast, to the previous analyses, the 2PL did not identify a significant mode effect on the test score level. However, it needs to be kept in mind that several items showed a rather poor fit to the 2PL in the first place which makes these analyses rather unstable. Moreover, the 2PL did not reflect the recommended scoring approach validated by the test authors.

# Additional References

Akaike, H. (1974). A new look at the statistical model identification. *IEEE Transactions on Automatic Control, 19*(6), 716–723. <https://doi.org/10.1109/TAC.1974.1100705>

Birnbaum, A. (1968). Some latent trait models and their use in inferring an examinee’s ability. In F. M. Lord & M. R. Novick (eds.), *Statistical Theories of Mental Test Scores* (pp. 397-472). Addison-Wesley Publishing.

Chalmers, R. P., & Ng, V. (2017). Plausible-value imputation statistics for detecting item misfit. *Applied Psychological Measurement, 41*, 372-387. <https://doi.org/10.1177/0146621617692079>

Chen W.-H., & Thissen D. (1997). Local dependence indexes for item pairs using item response theory. *Journal of Educational and Behavioral Statistics, 22*(3), 265-289. <https://doi.org/10.3102/10769986022003265>

Fong, C., Ratkovic, M., & Imai, K. (2021). *CBPS: Covariate Balancing Propensity Score*. R package version 0.22. <https://CRAN.R-project.org/package=CBPS>

Karabatsos, G. (2000). A critique of Rasch residual fit statistics. *Journal of Applied Measurement, 1*, 152-176.

Kolenikov, S. (2014). Calibrating survey data using iterative proportional ﬁtting (raking). *The Stata Journal, 14*(1), 22–59. <https://doi.org/10.1177/1536867X1401400104>

Kubinger, K. D., & Draxler, C. (2007). A comparison of the Rasch model and constrained item response theory models for pertinent psychological test data. In M. von Davier & C. H. Carstensen (Eds.), *Multivariate and mixture distribution Rasch models: Extensions and applications* (pp. 293-309). Springer.

Linacre, J. M. (2003). Size vs. significance: infit and outfit mean-square and standardized chi-square fit statistic. *Rasch Measurement Transactions, 17*(1), 918.

Linacre, J. M., & Wright, B., D. (1994). Chi-square fit statistics. *Rasch Measurement Transactions, 8*(2), 360.

Schwarz, G. E. (1978). Estimating the dimension of a model. *Annals of Statistics, 6*(2), 461–464. <https://doi.org/10.1214/aos/1176344136>

Smith, A. B., Rush, R., Fallowfield, L. J., Velikova, G., & Sharpe, M. (2008). Rasch fit statistics and sample size considerations for polytomous data. *BMC Medical Research Methodology, 8*, Article 33. <https://doi.org/10.1186/1471-2288-8-33>

Théberge, A. (2000). Calibration and restricted weights. *Survey Methodology, 26*(1)*,* 99–107.

Yen, W. M. (1993). Scaling performance assessments: strategies for managing local item dependence. *Journal of Educational Measurement, 30*, 187-213. [https://doi.org/10.1111/j.1745-3984.1993. tb00423.x](https://doi.org/10.1111/j.1745-3984.1993.%20tb00423.x)
